# Differential Proteoglycan Expression in Atherosclerosis Alters Platelet Adhesion and Activation

**DOI:** 10.3390/ijms25020950

**Published:** 2024-01-12

**Authors:** Amelia Drysdale, Maria Blanco-Lopez, Stephen J. White, Amanda J. Unsworth, Sarah Jones

**Affiliations:** 1 Department of Life Sciences, Manchester Metropolitan University, Manchester M1 5GD, UK; a.drysdale@mmu.ac.uk (A.D.); maria.r.blanco-lopez@stu.mmu.ac.uk (M.B.-L.); a.unsworth@mmu.ac.uk (A.J.U.); 2 Faculty of Medical Sciences, The Medical School, Newcastle University, Framlington Place, Newcastle upon Tyne NE2 4HH, UK; steve.white3@newcastle.ac.uk

**Keywords:** atherosclerosis, plaque rupture, plaque erosion, platelet, thrombosis, extracellular matrix, proteoglycan, collagen, biglycan, versican, decorin, hyaluronan

## Abstract

Proteoglycans are differentially expressed in different atherosclerotic plaque phenotypes, with biglycan and decorin characteristic of ruptured plaques and versican and hyaluronan more prominent in eroded plaques. Following plaque disruption, the exposure of extracellular matrix (ECM) proteins triggers platelet adhesion and thrombus formation. In this study, the impact of differential plaque composition on platelet function and thrombus formation was investigated. Platelet adhesion, activation and thrombus formation under different shear stress conditions were assessed in response to individual proteoglycans and composites representing different plaque phenotypes. The results demonstrated that all the proteoglycans tested mediated platelet adhesion but not platelet activation, and the extent of adhesion observed was significantly lower than that observed with type I and type III collagens. Thrombus formation upon the rupture and erosion ECM composites was significantly reduced (*p* < 0.05) compared to relevant collagen alone, indicating that proteoglycans negatively regulate platelet collagen responses. This was supported by results demonstrating that the addition of soluble biglycan or decorin to whole blood markedly reduced thrombus formation on type I collagen (*p* < 0.05). Interestingly, thrombus formation upon the erosion composite displayed aspirin sensitivity, whereas the rupture composite was intensive to aspirin, having implications for current antiplatelet therapy regimes. In conclusion, differential platelet responses and antiplatelet efficacy are observed on ECM composites phenotypic of plaque rupture and erosion. Proteoglycans inhibit thrombus formation and may offer a novel plaque-specific approach to limit arterial thrombosis.

## 1. Introduction

Atherosclerosis is a chronic, multi-decade inflammatory disease of blood vessels resulting in the development of an atherosclerotic plaque [1]. Plaque development is a complex process involving endothelial dysfunction, the development of intimal hyperplasia, the laying down of poorly organised and altered ECM, the trapping and oxidation of lipoproteins within the intima and the recruitment of inflammatory cells [2,3]. The acute disruption of atherosclerotic plaques through rupture or erosion exposes extracellular matrix (ECM) proteins. This triggers platelet adhesion and activation, propagating thrombus formation [4], which can lead to vascular occlusion and associated clinical manifestations, including myocardial infarction and stroke [5]. Evidence suggests that there are important differences in the composition of the exposed ECM following plaque rupture and erosion [6,7], which has the potential to differentially influence platelet responses and the thrombotic process observed for each plaque type. Indeed, differences have been reported in the structure and composition of thrombi associated with each plaque phenotype, with ruptured plaques more commonly resulting in occlusive thrombi with a significant red blood cell and fibrin component, while eroded plaques are less occlusive and are platelet-rich [8,9].

In addition to collagens, vascular proteoglycans are key components of the ECM [10]. Vascular proteoglycans play functional roles in modifying the biomechanical properties of fibrillar collagen, as well as regulating cell behaviour, platelet function and the development and progression of atherosclerotic plaques [3,11,12]. Proteoglycans identified at sites of atherosclerotic plaque rupture and erosion include biglycan, decorin, versican and high- and low-molecular-weight hyaluronans. Previous studies have described that proteoglycan abundance at the plaque–thrombus interface differs between ruptured and eroded plaques [6,13,14], with decorin and biglycan present in ruptured plaques and versican and hyaluronan in eroded plaques. Currently, it is unknown whether these variations in proteoglycan ECM composition play a role in platelet adhesion and activation and, therefore, the initiation and progression of thrombosis in each plaque type. 

Here, we present our findings, demonstrating that whilst type I and III collagens are the main platelet agonists present in ‘rupture’ and ‘erosion’ matrices, vascular proteoglycans play differential roles in regulating platelet–collagen interactions, modulating adhesion and regulating thrombus formation.

## 2. Results 

### 2.1. Recombinant Vascular Proteoglycans Support Platelet Adhesion and Spreading

**Figure 1:**
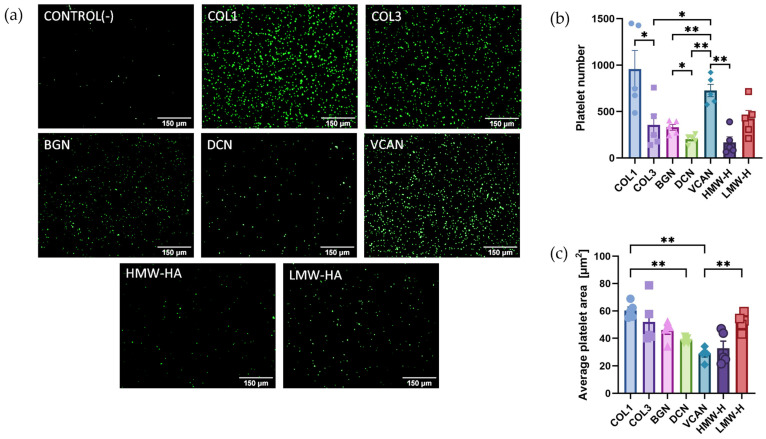
Platelet adhesion and spreading on different extracellular matrices. (**a**) Platelets fluorescently labelled with DIOC6 were incubated in 96-well plates coated with either 20 µg/mL collagen I (COL1); collagen III (COL3); Biglycan (BGN); Decorin (DCN); Versican (VCAN); high-molecular-weight hyaluronan (HMW-HA); low-molecular-weight hyaluronan (LMW-HA) or goat serum blocking buffer (CONTROL) for 45 min at 37 °C. Following washing, (**b**) the number of platelets remaining and (**c**) the average platelet area was quantified as a measure of platelet adhesion and spreading, respectively. Data are presented as mean ± SEM, (*n* = 5), * *p* < 0.05, ** *p* < 0.01.

Variations in platelet responses to different vascular proteoglycans may explain the observed differences in thrombus formation in patients who experience plaque rupture or plaque erosion. However, there is a lack of studies directly comparing platelet interactions with different proteoglycans. Therefore, platelet static adhesion assays were performed to determine ligand binding affinity to different ECM proteins and to assess proteoglycan-initiated platelet spreading. Washed platelets (2 × 10^7^/mL) were allowed to adhere to 20 µg/mL of either type I and III collagens, recombinant biglycan, decorin, versican or HMW and LMW hyaluronan for 45 min at 37 °C before fixation and visualisation. The results demonstrated that the number of adhered platelets was significantly higher in the case of type I collagen than in type III collagen (Figure 1). The most adhesive vascular proteoglycan under static conditions was versican, with significantly higher platelet adhesion than decorin (*p* < 0.01), biglycan (*p* < 0.01), HMW hyaluronan (*p* < 0.01) and type III collagen (*p* < 0.05) (Figure 1a). Average platelet area, a marker of platelet spreading, was highest in the case of type I collagen compared with decorin (*p* < 0.01) and versican (*p* < 0.01). These initial observations demonstrate that platelet adhesion and spreading to individual ECM components varies significantly amongst collagens and vascular proteoglycans. 

### 2.2. Vascular Proteoglycans Do Not Stimulate Platelet Activation Responses

**Figure 2:**
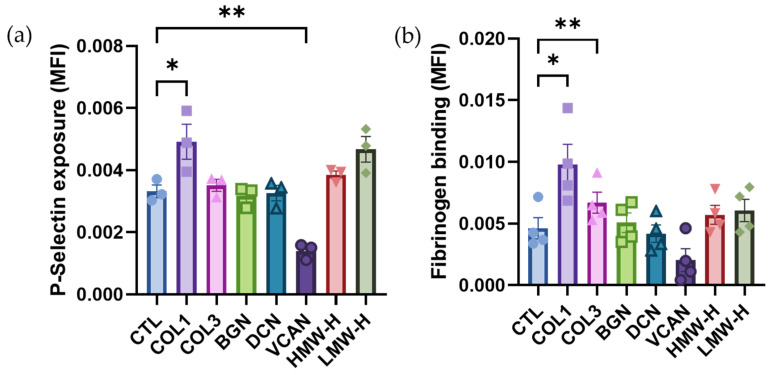
Platelet activation and fibrinogen binding. Platelets were stimulated with 20 µg/mL collagen I (COL1); collagen III (COL3); Biglycan (BGN); Decorin (DCN); Versican (VCAN); high-molecular-weight hyaluronan (HMW-HA); low-molecular-weight hyaluronan (LMW-HA) or vehicle control and (**a**) surface P-selectin levels (*n* = 3) and (**b**) fibrinogen binding (*n* = 4) were measured using flow cytometry. Data are presented as mean ± SEM, * *p* < 0.05, ** *p* < 0.01.

To determine whether vascular proteoglycans can stimulate platelet activation, flow cytometry was performed to investigate platelet alpha granule secretion (P-Selectin exposure) and integrin αIIbβ3 activation (Fibrinogen binding) in response to the different proteoglycans as well as type I and III collagen. The results demonstrated that at the concentrations tested (20 µg/mL), type I collagen (*p* < 0.05) was the only ECM component that stimulated an increase in P-selectin surface exposure compared to unstimulated control (Figure 2a), while both type I (*p* < 0.05) and type III collagens (*p* < 0.01) increased platelet fibrinogen binding (Figure 2b). In comparison, none of the vascular proteoglycans investigated promoted P-selectin exposure or fibrinogen binding. These findings suggest that vascular proteoglycans do not play a significant role in the initiation of platelet functional responses. 

### 2.3. Vascular Proteoglycans Alone Do Not Support Robust Thrombus Formation under Arterial and Elevated Flow

**Figure 3:**
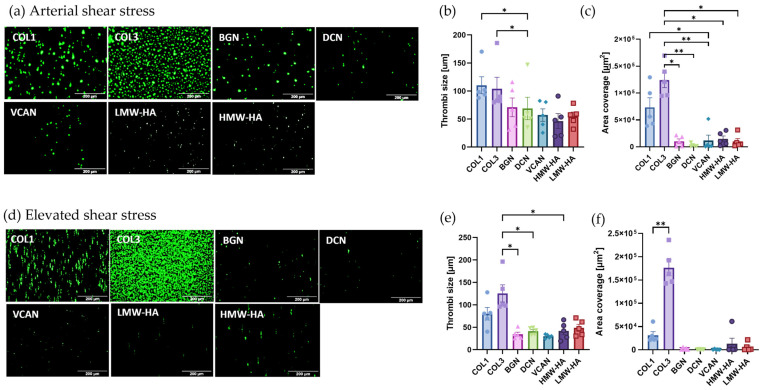
Thrombus formation on individual extracellular matrix proteins under arterial and elevated shear stress. Whole blood labelled with DIOC6 was perfused through ibidi µ-slides coated with 100 µg/mL type 1 collagen (COL1); type III collagen (COL3); versican (VCAN); biglycan (BGN); decorin (DCN); high-molecular-weight hyaluronan (HMW) or low-molecular-weight hyaluronan; (LMW) at (**a**–**c**) arterial shear stress (15 dynes/cm^2^) or (**d**–**f**) elevated shear stress (75 dynes/cm^2^). Platelets were visualised using fluorescence microscopy, and thrombi size at (**b**) arterial and (**e**) elevated shear stress and platelet area coverage at (**c**) arterial and (**f**) elevated shear stress were quantified using Fiji/ImageJ. Data represent mean ± SEM; *n* = 5; * *p* < 0.05; ** *p* < 0.01.

To further investigate the interactions between platelets and different proteoglycans, in vitro thrombus formation experiments were performed at arterial shear stress (15 dynes/cm^2^) and elevated shear stress (75 dynes/cm^2^) to mimic the vascular environment of stenosed coronary vessels [15]. Thrombus formation on type I and III collagen was included as a comparative control. As expected, platelets formed robust thrombi on both type I and type III collagen under both arterial and elevated shear, with significantly higher amounts of thrombus formation on type III vs. type I collagen under elevated shear (Figure 3a–c vs. Figure 3d,e). In comparison to the collagens, the single vascular proteoglycans were unable to support robust thrombus formation with significantly lower levels of platelet adhesion and thrombus formation observed under both arterial and elevated shear. No significant differences in thrombus formation were observed between the different vascular proteoglycans tested under both shear conditions. 

### 2.4. Vascular Proteoglycans Regulate Platelet Responses to Collagen

**Figure 4:**
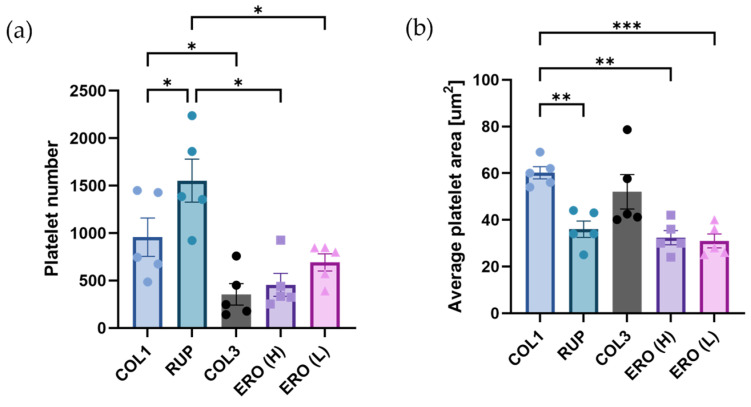
Platelet adhesion and spreading on type I and type III collagen compared to ‘rupture’ and ‘erosion’ composite matrices. Washed platelets were incubated for 45 min at 37 °C in wells coated with type I collagen (COL1; 20 µg/mL), type III collagen (COL3; 20 µg/mL), a ‘rupture’ composite (RUP) containing type I collagen, biglycan and decorin (20 µg/mL of each protein) or erosion composites (ERO) consisting of type III collagen, versican and high-molecular-weight hyaluronan (H) or low-molecular-weight hyaluronan (L) (20 µg/mL each). (**a**) Platelets were visualised using fluorescence microscopy, and (**a**) platelet number and (**b**) platelet area were quantified using Fiji/ImageJ. Data represent mean ± SEM. *n* = 5; * *p* < 0.05; ** *p* < 0.01; *** *p* < 0.001.

Investigations have so far focused on the contribution of single proteoglycans to platelet adhesion and activation. However, in vivo, platelets are exposed to a combination of collagens and proteoglycans, which have the potential to regulate the activity of one another. Disease-relevant composite matrices were therefore constructed to represent the combinations of ECM exposed following plaque rupture (type I collagen, biglycan and decorin) and plaque erosion (type III collagen, versican and either HMW or LMW hyaluronan) [6,9]. Under static conditions, platelet binding was higher on the ‘ruptured’ matrix compared to both erosion composites and collagen I or III alone (Figure 4a, *p* < 0.05). However, platelet spreading (average platelet area) was significantly greater on Type I collagen compared with both ‘rupture’ (*p* < 0.01) and ‘erosion’ (*p* < 0.01; *p* < 0.001) matrices. Interestingly, no significant differences were observed between ‘erosion’ composites containing HMW and LMW hyaluronan (Figure 4).

### 2.5. Composite ‘Rupture’ and ‘Erosion’ Matrices Are Less Thrombogenic than Collagen under Shear

**Figure 5:**
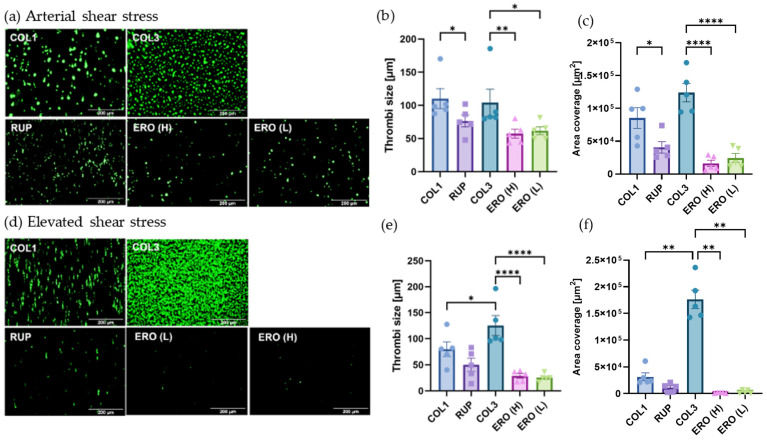
Thrombus formation on disease-relevant matrix composites using arterial and elevated shear stress. Whole blood labelled with DIOC6 was perfused through Ibidi µ-slides coated with 100 µg/mL type I collagen (COL1), type III collagen (COL3), a ‘rupture’ composite (type I collagen, biglycan and decorin; RUP) or erosion composites (ERO) consisting of type III collagen, versican and high-molecular-weight hyaluronan (H) or low-molecular-weight hyaluronan (L) at (**a**–**c**) arterial (15 dynes/cm^2^) and (**d**–**f**) elevated (75 dynes/cm^2^) shear stress. Chambers were imaged. Platelets were visualised using fluorescence microscopy, and thrombi size at (**b**) arterial and (**e**) elevated shear stress and platelet area coverage at (**c**) arterial and (**f**) elevated shear stress were quantified using Fiji/ImageJ. Data represent mean ± SEM; *n* = 5; * *p* < 0.05; ** *p* < 0.01, **** *p* <0.0001.

To explore the relevance of ECM composition in arterial thrombosis under conditions of physiological and pathological elevated shear, thrombus formation on ‘rupture’ and ‘erosion’ matrices were compared under arterial (15 dynes/cm^2^; Figure 5a–c) and elevated (75 dynes/cm^2^; Figure 5d,e) shear stress using an in vitro model of thrombus formation. Type I and type III collagen were included as comparative controls. 

Under arterial shear stress conditions, thrombus size (Figure 5b) and platelet area coverage (Figure 5c) were significantly reduced on the rupture matrix compared to type I collagen alone (*p* < 0.05). This significant difference was lost under elevated shear stress (Figure 5e,f). Interestingly, differences in thrombus formation were observed between both ‘erosion’ matrices and type III collagen alone under arterial shear and elevated shear stress, with both thrombus size and platelet area coverage significantly reduced. Taken together, these findings indicate that thrombus formation on composites representing plaque rupture and erosion support platelet adhesion and aggregation but at levels significantly lower than those observed using type I or type III collagen.

### 2.6. Aspirin Efficacy Is Altered on Composite ‘Rupture’ and ‘Erosion’ Matrices

**Figure 6:**
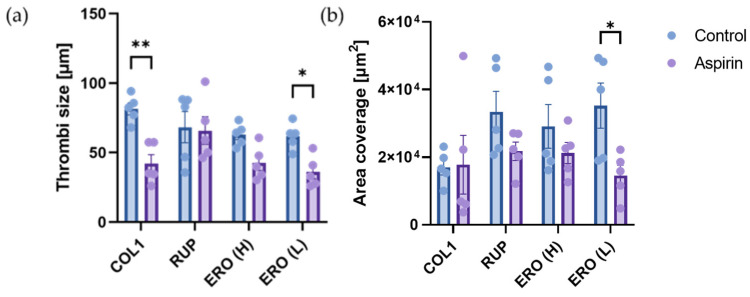
Thrombus formation in the presence of aspirin. Whole blood labelled with DIOC6 was perfused through ibidi µ-slides coated with 100 µg/mL type I collagen (COL1), a ‘rupture’ composite (type I collagen, biglycan and decorin; RUP) or erosion composites (ERO) consisting of type III collagen, versican and high-molecular-weight hyaluronan (H) or low-molecular-weight hyaluronan (L) at arterial (15 dynes/cm^2^) shear stress. Chambers were imaged. Platelets were visualised using fluorescence microscopy, and (**a**) thrombi size and (**b**) platelet area coverage were quantified using Fiji/ImageJ. Data represent mean ± SEM; *n* = 5; * *p* < 0.05; ** *p* < 0.01.

There is currently no patient stratification based on plaque phenotype, and it is not known whether aspirin is as effective following both plaque rupture and erosion. Therefore, we investigated whether the efficacy of aspirin to reduce thrombus formation in vitro under arterial shear was altered depending on whether the trigger was a ‘rupture’ or ‘erosion’ plaque composite. As shown in Figure 6, aspirin significantly reduced thrombus size on type I collagen (*p* < 0.01) but not area coverage. Aspirin also significantly reduced thrombus size (*p* < 0.05) and area coverage (*p* < 0.05) on the erosion composite containing LMW-hyaluronan. In contrast, aspirin had no significant effect on thrombus size or area coverage, on the rupture composite. These results demonstrate the differential efficacy of antiplatelet therapy depending on plaque phenotype, with limited effects of aspirin demonstrated in rupture phenotypes under arterial shear stress.

### 2.7. Biglycan and Decorin Exhibit Antithrombotic Effects by Limiting Thrombus Formation on Type I Collagen

**Figure 7:**
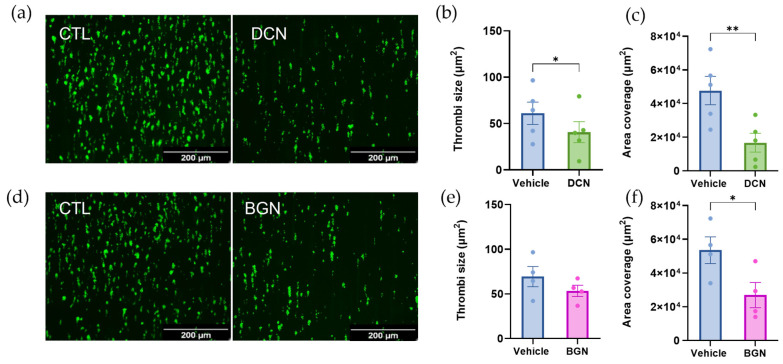
Thrombus formation on type 1 collagen with the addition of soluble decorin (DCN) or biglycan (BGN). Whole blood labelled with DIOC6 was perfused through ibidi µ-slides coated with 100 µg/mL type I collagen (COL1) at arterial shear stress (15 dynes/cm^2^) following the addition of recombinant (**a**–**c**) decorin (DCN) or (**d**–**f**) biglycan (BGN) (20 µg/mL) to the whole blood. Platelets were visualised using fluorescence microscopy, and (**b**,**e**) thrombi size and (**c**,**f**) platelet area coverage were quantified using Fiji/ImageJ. Data represent mean ± SEM; *n* = 4; * *p* < 0.05; ** *p* < 0.01.

To investigate whether soluble biglycan or decorin could act as antiplatelet agents, thrombus formation assays were performed on type I collagen under arterial shear stress following the addition of recombinant biglycan (20 µg/mL) or decorin (20 µg/mL) to the whole blood (Figure 7). Decorin was shown to significantly reduce thrombus size (Figure 7b; *p* < 0.05) and area coverage (Figure 7c; *p* < 0.01). Similarly biglycan significantly reduced area coverage (Figure 7f; *p* < 0.05), indicating reduced platelet adhesion. These findings support our observations that vascular proteoglycans can modulate platelet responses to collagen, limiting platelet adhesion and activation. 

## 3. Discussion

The disruption of atherosclerotic plaques by either rupture or erosion exposes platelets to different concentrations and combinations of ECM components, potentially changing both platelet adhesion and activation, with consequences for thrombotic occlusion and patient management. Proteoglycans represent a major component of the ECM [10], and studies have shown differential expression of proteoglycans depending on plaque phenotype [6]. The purpose of this study was to investigate platelet responses to vascular proteoglycans relevant to the development and progression of atherosclerosis and to elucidate whether differential proteoglycan expression altered platelet functional responses. Platelet adhesion and thrombus formation evoked by individual proteoglycans and combinations of proteoglycans and collagens designed to represent key differential ECM components of ruptured and eroded plaques were investigated. Importantly, we performed experiments under static conditions (of relevance in occluded vessels), under normal physiological flow and at elevated flow that corresponds to the mean shear stress observed on eroded plaques [15]. The results demonstrated that while proteoglycans support platelet adhesion under static conditions, platelet spreading and activation were limited. Under physiological and elevated flow, proteoglycans had limited capacity to adhere platelets and initiate thrombus formation. They also appeared to modulate collagen-induced thrombus formation, demonstrating differing effects of the proteoglycans to thrombosis, and highlighting the importance of the experimental design. 

Static adhesion experiments demonstrated that platelets could adhere to proteoglycans; however, the levels of adhesion were significantly lower than type I collagen, except for versican, where high levels of adhesion were observed. The adhesive properties of versican were not sustained at arterial or elevated shear stress conditions, which is consistent with previous reports demonstrating specific platelet interactions with versican at low but not high shear rates [16]. Platelet adhesion and thrombus formation for the other proteoglycans were also limited at arterial and elevated levels of shear stress, with significantly larger and more abundant thrombi formed on the collagens. These findings can be attributed to the different receptors involved. Biglycan and decorin both bind to platelets via integrin α2β1 [17], while versican and hyaluronan interact with platelets via surface P-Selectin and CD44, respectively [16,18,19]. These receptors primarily regulate platelet adhesion, not activation, as observed in flow cytometry experiments where the proteoglycans failed to evoke alpha granule secretion or fibrinogen binding (Figure 2). In contrast, the collagen GPVI receptor initiates platelet activation pathways, resulting in the conformational change of integrin αIIbβ3, platelet aggregation, and thrombus formation. 

Interestingly, flow cytometry results indicated that versican not only failed to activate platelets but also reduced resting levels of surface P-Selectin. Versican is a large proteoglycan, with the core protein having a molecular weight of 370 kDa, in addition to 12–15 GAG chains of approximately 45 kDa each [20,21]. Therefore, it is possible that the interaction of versican with platelet P-Selectin may sterically hinder antibody binding and detection in flow cytometry experiments rather than reduce cell surface levels. Similarly, there is the potential that versican binding to platelet P-selectin could reduce interactions with its physiological ligand P-selectin glycoprotein ligand -1 (PSGL-1), which is expressed on endothelial cells and inflammatory cells, and negatively regulates platelet contributions to thrombosis and inflammation.

Investigations into platelet adhesion and thrombus formation under flow demonstrated very little platelet adhesion and thrombus formation in response to the proteoglycans or hyaluronan compared to type I and III collagen. Interestingly, the differential platelet responses to type I collagen and type III collagen were observed with significantly more platelets adhered to type III collagen under elevated shear stress than type I collagen. This could, in part, be due to the nature of the collagens used. Type 1 Horm collagen is fibrillar, and platelets bind along the fibrillar stands [22], whereas the soluble type III collagen likely provides a more even coverage of the flow chambers. In addition, VWF, which facilitates the initial platelet interaction between collagen and platelet GPIb, is dependent on high shear to unravel the large VWF multimers and has a higher affinity for type III collagen [23]. Type III collagen and elevated shear stress are particularly relevant in plaque erosion [6,9,15], which represents a growing proportion of myocardial infarctions, due to better management of risk factors for plaque rupture through the use of statins, reducing lipid levels and inflammation [24,25]. 

Advances in intravascular imaging have now made it possible to stratify patients based on plaque phenotype using optical coherence tomography (OCT) [26]; however, there is a paucity of studies investigating antithrombotic efficacy based on plaque phenotype, limiting translation to personalised antithrombotic treatment. The development of novel plaque-specific antithrombotics is also hindered by the lack of experimental plaque erosion models. In this study, we developed novel composites to represent the key differences in ECM exposed in ruptured or eroded plaques based on work conducted by Kolodgie et al. [6]. Platelet responses were compared between the plaque phenotypes, including the relevant individual collagen. The ‘rupture’ composite contained type I collagen, biglycan and decorin, whilst the ‘erosion’ composite consisted of type III collagen, versican and either HMW or LMW hyaluronan. The addition of biglycan and decorin to type I collagen to form the ‘rupture’ matrix significantly reduced platelet adhesion and the size of thrombi that formed in the chambers despite the concentration of collagen remaining the same. These results are supported by previous studies suggesting that proteoglycan receptor binding predominantly mediates platelet adhesion and that the competitive binding of integrin αIIβ1 may limit type I collagen activity in the ‘rupture’ composite [27]. Platelet adhesion and thrombus formation on ‘erosion’ matrices were significantly reduced compared with type III collagen, demonstrating a role for hyaluronan and versican in the regulation of platelet interactions with type III collagen under flow and versican’s ability to limit P-selectin interactions. The propensity for clinical presentation of plaque rupture or erosion may, therefore, depend to some degree on the relative abundance of different proteoglycans within the plaque, in addition to the pro-coagulant status of the patient at the time of plaque disruption. 

Results indicating that platelet responses are altered when collagens are exposed in the presence of vascular proteoglycans, such as those found in both plaque rupture and plaque erosion, may have implications on antiplatelet efficacy, which is routinely investigated in vitro using type I collagen in isolation [28,29,30,31,32]. The differences observed between ‘rupture’ and ‘erosion’ matrices also indicated that different mechanisms may be involved in the formation of arterial thrombi; therefore, the efficacy of antithrombotic drugs may differ depending on plaque type. To investigate this further, blood was pre-treated with aspirin before being perfused through chambers coated with type I collagen or the ‘plaque’ and ‘erosion’ composites at an arterial shear rate. As expected, aspirin significantly reduced the size of thrombi formed on type I collagen, but not the area coverage, consistent with its actions blocking secondary signaling through the inhibition of thromboxane synthesis but with no effects on platelet adhesion. Aspirin also successfully reduced the size of the thrombi generated on both erosion matrices. Erosion composites containing LMW hyaluronan were particularly sensitive to aspirin. In monocytes, LMW hyaluronan stimulates phospholipase A2 activation and arachidonic acid release [33], the rate-limiting step in thromboxane A2 generation; this may explain the increased aspirin sensitivity if similar signaling effects are observed in platelets. In contrast, aspirin did not significantly reduce the size of thrombi, which developed on the ‘rupture’ matrices, highlighting plaque-specific differences to antiplatelet drugs. These findings suggest that aspirin may not be the most appropriate choice of antiplatelet prophylaxis for those patients at risk of plaque rupture and may explain why aspirin only offers a 20% reduction in the risk of secondary thrombotic events [34]. 

Given that GPVI is the most thrombogenic component of the ECM [35,36] and is less important for haemostasis [37], recent advances in antiplatelet therapy have focused on disrupting the interaction between platelet GPVI and type I collagen. Drugs, including Revacept, a fusion protein that competes with endogenous GPVI for collagen [38] and Glenzocimab, a humanised fab fragment that blocks GPVI from interacting with collagen [39], have entered clinical trials [9]. Both have demonstrated good safety profiles [38,40]. In the ISAR-PLASTER Phase 2 clinical trial, Revacept showed no increase in bleeding in patients undergoing elective percutaneous coronary intervention (PCI); however, it did not reduce the incidence of myocardial infarction [38]. Glenzocimab is currently in a phase 2 clinical trial for acute ischemic stroke (ACTIMIS and ACTISAVE) and is due to enter a phase 2b clinical trial (LIBERATE) for myocardial infarction. The results presented in this study highlight that recombinant proteoglycans may offer a similar approach. Biglycan [41] and decorin [42] both bind type I collagen, and the presence of biglycan and decorin in a matrix together with type I collagen reduced collagen I-induced thrombus formation. Similarly, versican and hyaluronan reduced thrombus formation on type III collagen. The potential of recombinant proteoglycans to inhibit thrombus formation when administered directly to whole blood was demonstrated with soluble biglycan and decorin, both of which significantly reduced thrombus formation on type I collagen. However, further work is required to determine whether the proteoglycans bind platelet receptors (e.g., α2β1) or the immobilised collagen to reduce platelet–collagen interaction. The benefits of proteoglycans in preventing thrombus formation have been presented in several other studies. Decorin mimetic (DS-SILY), for example, when administered directly to balloon-injured arteries in miniature pigs, demonstrated markedly reduced platelet adhesion to the vascular wall following endothelial denudation [43]. Similarly, biglycan has been shown to be protective in a mouse model of atherosclerosis, where APOE-deficient mice, also deficient in biglycan, displayed enhanced circulating thrombin levels, elevated platelet activation and increased adhesion in vivo compared to control APOE knockouts [44]. Perlecan, a proteoglycan also found in the vascular wall, has recently been shown to induce platelet activation under static conditions but inhibits collagen-induced thrombus formation under arterial shear stress conditions [45]. The interest in proteoglycans as novel therapeutic agents has gained traction over recent years [46]. Improved technologies to create recombinant proteoglycans, in addition to the development of a number of mimetics, have accelerated our understanding of their biological roles, with a wide range of potential therapeutic applications being tested [46]. The results here indicated that arterial thrombosis is an additional application where proteoglycan therapeutics may be of benefit.

In conclusion, platelet adhesion, thrombus formation and aspirin sensitivity vary depending on whether the ECM exposed to platelets is phenotypic of ‘ruptured’ or ‘eroded’ plaques. Proteoglycans appear to negatively regulate thrombus formation, dampening platelet responses to collagen when co-expressed in the matrices. The addition of biglycan or decorin directly to whole blood substantially reduces thrombus formation on type I collagen, indicating that recombinant proteoglycans or proteoglycan mimetics may offer a novel therapeutic strategy to directly inhibit platelet–plaque interactions without compromising haemostasis. Further work is required to determine the mechanisms by which proteoglycans modulate thrombus formation and to assess whether the inhibition evoked by different proteoglycans is collagen-type-specific; and could therefore, be tailored to patients depending on their plaque phenotype.

## 4. Materials and Methods 

### 4.1. Materials 

Type I collagen was purchased from Labmedics (Abingdon, UK), type III collagen and versican were obtained from Cambridge Bioscience (Cambridge, UK), and biglycan was obtained from Caltag MedSystems (Buckingham, UK). Decorin, high-molecular-weight (HMW) hyaluronan, low-molecular-weight (LMW) hyaluronan and aspirin were purchased from Bio-Techne (Abingdon, UK). 3,3′-Dihexyloxacarbocyanine iodide (DIOC6), phosphate-buffered saline (PBS) and buffer reagents for Tyrodes-HEPEs buffer and acid citrate dextrose (ACD) were purchased from Sigma Aldrich (Poole, UK). Mouse anti-human PE-Cy5 CD62P antibody was purchased from BD Biosciences (Berkshire, UK), and rabbit anti-human FITC fibrinogen was obtained from Agilent (London, UK). All other reagents were obtained from previously described sources.

### 4.2. Platelet Preparation

For experiments using human platelets, blood was obtained from consenting aspirin-free healthy volunteers following procedures approved by the Manchester Metropolitan University Research Ethics Committees (EthOS reference 12762). Peripheral blood was taken from healthy male and female volunteers aged 18–65 into 3.2% (*v*/*v*) sodium citrate BD vacutainers (Bunzl Healthcare, Manchester, UK). The blood was centrifuged at 100× *g* for 20 min at room temperature to isolate platelet-rich plasma. To prepare ADP-sensitive washed platelets, 1:8 *v/v* acid citrate dextrose (ACD; 29.9 mM trisodium citrate, 113.8 mM glucose, 2.9 mM citric acid, pH 6.4) was added to PRP before centrifugation at 350× *g* for 20 min at room temperature. Platelet-poor plasma was discarded, and the platelet pellet was then resuspended in Tyrodes-HEPES buffer (134 mM sodium chloride, 2.9 mM potassium chloride, 0.34 mM sodium phosphate dibasic, 12 mM sodium bicarbonate, 20 mM HEPES, 1 mM magnesium chloride, pH 7.3) with added glucose (5 mM) to the required density of 2 × 10^7^ platelets/mL for adhesion and spreading assays.

### 4.3. Platelet Adhesion and Spreading Assays

Washed platelets (2 × 10^7^ platelets/mL) were added to wells of a 96-well plate coated with 20 µg/mL of type I collagen (Equine tendon-derived), type III collagen, biglycan, decorin, versican, high-molecular-weight (HMW) hyaluronan or low-molecular-weight (LMW) hyaluronan alone or in combination and left to adhere and spread for 45 min at 37 °C. The wells were then washed twice gently with phosphate-buffered saline (PBS) to remove non-adherent platelets and samples fixed in 4% (*w*/*v*) paraformaldehyde (PFA) for 10 min. Following a further wash with PBS, 1 μM 3,3′-dihexyloxacarbocyanine iodide (DIOC6) was added to the platelets to enable visualisation and incubated for 30 min at room temperature. The wells were washed well with PBS and imaged using a Celena S Logos fluorescent digital imaging system and 10× lens. The experiments were also performed using combinations of proteins to represent the predominant ECM proteins exposed to blood following plaque rupture (type I collagen, biglycan and decorin) and plaque erosion (type III collagen, versican and hyaluronan). The composites used in static adhesion and spreading experiments contained 20 µg/mL of each protein. 

### 4.4. Flow Cytometry Analysis of Platelet Activation Markers

Platelet activation was assessed via the measurement of P-Selectin exposure and fibrinogen binding. Tyrodes-HEPES buffer containing 20 μg/mL ECM proteins was added to wells of a flat bottom 96-well plate with, PRP (1:10 dilution) and either mouse anti-human PE-Cy5 CD62P (1:20) or rabbit anti-human FITC fibrinogen (1:20 dilution). The plate was incubated in the dark for 30 min, and the reaction was stopped by the addition of 2% (*w*/*v*) PFA to fix the samples. The samples were then analysed using a Miltenyi Biotec MACSQuant flow cytometer. The platelets were gated based on forward and side scatter profiles, with 10,000 events counted per gate. The median fluorescence intensity of the positive platelet population was then calculated using FlowJo software version 10.7.1 (BD Biosciences, Berkshire, UK).

### 4.5. In Vitro Thrombosis Model 

Ibidi VI 0.1 µ-slides (Thistle Scientific, Rugby, UK) were used in a high-throughput model setup to measure thrombus formation on ECM proteins, enabling six chambers to be assessed under flow at one time. Briefly, the slide chambers were coated with 100 μg/mL type I collagen, type III collagen, biglycan, decorin, versican, high-molecular-weight (HMW) hyaluronan or low-molecular-weight (LMW) hyaluronan for one hour at RT prior to performing the experiments. In experiments where composites were used to represent plaque rupture (type I collagen, biglycan and decorin) or plaque erosion (type III collagen, versican and hyaluronan), 100 µg/mL of each protein was used. 

The thrombus formation model was set up using a six-channel programmable syringe pump (World Precision Instruments, Hitchin, UK) to withdraw the blood through the channels at defined shear stress to represent arterial conditions (15 dyne/cm^2^) and stenotic conditions (75 dyne/cm^2^) where elevated shear stress is observed. Prior to perfusion, whole blood was incubated with 1 µM DIOC6 for 15 min at room temperature to enable visualisation of the platelets. In experiments using aspirin, whole blood was incubated with 30 μM aspirin or vehicle control (0.03% ethanol) at 37 °C for 30 min prior to perfusion. Similarly, in experiments where proteoglycans were added to the blood, decorin (20 µg/mL), biglycan (20 µg/mL) or vehicle control (PBS) was added 30 min prior to perfusion. The blood was perfused through the chambers for 7 min, and thrombus formation was imaged at 488 nm using a Celena S Logos fluorescent digital imaging system. The average thrombi size and area coverage were analysed using Fiji/ImageJ thresholding. 

### 4.6. Statistical Analysis

Statistical analysis was performed using GraphPad Prism 9 (Graphpad, La Jolla, CA, USA). A normal distribution of the data was confirmed using a Shapiro–Wilk test, and statistical significance was determined using paired or unpaired one-way ANOVAs (with Tukey post hoc correction for multiple comparisons). Where two groups were compared, Student’s *t*-tests were performed. Fiji/ImageJ thresholding was used for microscopy and image analysis. Data are expressed as mean ± standard error of the mean (SEM), and *p* values < 0.05 are considered significant. 

## Data Availability

The data presented in this study are available on request from the corresponding author.
